# Ca^2+^/Calmodulin Binding to PSD-95 Downregulates Its Palmitoylation and AMPARs in Long-Term Depression

**DOI:** 10.3389/fnsyn.2019.00006

**Published:** 2019-03-12

**Authors:** Dhrubajyoti Chowdhury, Johannes W. Hell

**Affiliations:** Department of Pharmacology, University of California, Davis, Davis, CA, United States

**Keywords:** PSD-95, palmitoylation, AMPAR, LTD, synapse

## Abstract

AMPA-type glutamate receptors (AMPARs) are clustered into functional nanodomains at postsynaptic sites through anchorage by the scaffolding protein, postsynaptic density protein-95 (PSD-95). The synaptic abundance of AMPARs is dynamically controlled in various forms of synaptic plasticity. Removal of AMPARs from the synapse in long-term depression (LTD) requires mobilization of PSD-95 away from the synapse. The molecular mechanisms underlying PSD-95 dispersal from the synapse during LTD are not completely understood. Here we show that, following Ca^2+^ influx, binding of Ca^2+^/calmodulin (CaM) to PSD-95 triggers loss of synaptic PSD-95 as well as surface AMPARs during chemically induced LTD in cultured rat neurons. Our data suggest that a reduction in PSD-95 palmitoylation mediates the effect of Ca^2+^/CaM on PSD-95 synaptic levels during LTD. These findings reveal a novel molecular mechanism for synaptic AMPAR regulation in LTD.

## Introduction

Activity-dependent changes in the abundance of postsynaptic AMPA-type glutamate receptors (AMPARs) lie at the core of various long-lasting forms of synaptic plasticity (Henley and Wilkinson, 2016). One such form of plasticity, long-term depression (LTD) involves removal of AMPARs from the synapse via molecular mechanisms that are incompletely understood (Collingridge et al., 2010). Through interaction of their auxiliary subunits, AMPARs are anchored to postsynaptic sites by scaffold proteins of which PSD-95 is the most abundant (Chen et al., 2005). Consisting of three PSD-95/Discs large/zona occludens-1 (PDZ) domains, followed by a Src-homology-3 domain (SH3) and a catalytically inactive guanylate kinase (GK) domain, PSD-95 not only stabilizes ionotropic glutamate receptors at the synapse but also couples them to signaling enzymes, and adaptors (Won et al., 2017). Based on experimental work using transgenic animals, overexpression, knockdown and molecular replacement strategies, PSD-95 has been proposed to serve as a “slot” protein for synaptic AMPARs with PSD-95 abundance at the synapse determining synaptic AMPAR content (Schnell et al., 2002; Beique and Andrade, 2003; Stein et al., 2003; Beique et al., 2006). Changes in levels of PSD-95 directly influence the levels of synaptic AMPARs and provide a key molecular substrate for NMDAR-dependent synaptic plasticity, including LTD. Therefore, elucidating the molecular mechanisms of activity-driven changes in PSD-95 synaptic localization is fundamental to understand how synaptic plasticity at excitatory synapses is achieved.

Several activity-dependent post-translational modifications (PTMs) of PSD-95 such as palmitoylation, ubiquitination and phosphorylation at multiple sites have been reported to dynamically regulate synaptic PSD-95 levels in response to LTD-inducing stimuli in the form of glutamate receptor agonists (El-Husseini Ael et al., 2002; Colledge et al., 2003; Kim et al., 2007; Nelson et al., 2013). Recently, PSD-95 has been identified as a substrate for Lys63(K63)-linked polyubiquitination that is associated with NMDA-induced PSD-95 declustering at synapses (Ma et al., 2017). In addition, conjugation of the ubiquitin-like protein, Nedd8, to PSD-95 is required for dendritic spine maturation (Vogl et al., 2015). PSD-95 represents a major palmitoylated protein in neurons and palmitoylation of PSD-95 at Cys 3 and Cys 5 within its N-terminal domain is essential for its postsynaptic targeting (Topinka and Bredt, 1998; Craven et al., 1999). Palmitoylation is the addition of the saturated 16-carbon fatty acid, palmitic acid, to specific cysteine residues of substrate proteins by thioester linkage. This common post-translational lipid modification is uniquely reversible and allows proteins to shuttle between intracellular compartments and relocalize in response to physiological signals (Fukata and Fukata, 2010). Upon glutamate receptor stimulation, accelerated depalmitoylation of PSD-95 led to diffusion of PSD-95 away from postsynaptic sites accompanied by AMPAR endocytosis, thus downregulating synaptic strength similar to LTD (El-Husseini Ael et al., 2002). Previous work from our group has shown that Ca^2+^/calmodulin (CaM) binding to the PSD-95 N-terminus antagonizes palmitoylation and promotes release of PSD-95 from synapses upon both acute and chronic increases in synaptic activity (Zhang et al., 2014; Chowdhury et al., 2018). In the present study, we investigated whether this mechanism contributes to the loss of synaptic PSD-95 and AMPARs in response to an LTD-inducing stimulus.

Here we report that PSD-95 palmitoylation is reduced upon chemically inducing LTD in cultured neurons. Using a molecular replacement strategy, we found that CaM binding to Glu 17 within the PSD-95 N-terminus is required for this reduction in PSD-95 palmitoylation and, as a consequence, dispersal of PSD-95 from the synapse and downregulation of surface AMPARs in LTD. Hence, this study delineates a precise molecular mechanism for NMDAR-dependent LTD.

## Results

### Chemically Induced LTD Reduces PSD-95 Palmitoylation

**FIGURE 1:**
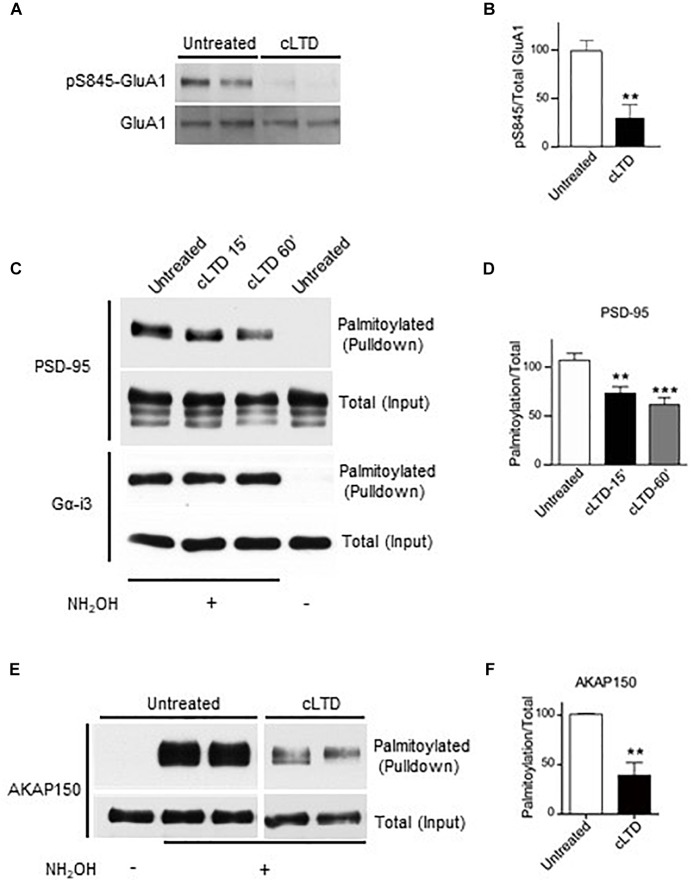
Effect of chemical LTD (cLTD) treatment on palmitoylation of endogenous PSD-95 and AKAP150. Cultured cortical neurons at DIV17 were either left untreated or treated with NMDA (50 μM) for 5 min followed by washout for 15 min (cLTD) before harvesting, extraction, and analysis of palmitoylation by ABE method and pulldown with NeutrAvidin agarose beads. **(A)** Total lysates were analyzed by immunoblotting to examine levels of S845 phosphorylation by immunoblotting with a phospho-specific antibody against phosphoS845. After stripping, the same blot was reprobed with an antibody against total GluA1. Representative immunoblots are shown. **(B)** Quantification of GluA1 S845 phosphorylation normalized to total GluA1 levels per condition. (^∗∗^*p* < 0.01; *t*-test, *n* = 4–5 per condition from three independent sets of cultures). Untreated condition was set to equal 100%. **(C,E)** Representative immunoblots of NeutrAvidin pulldown samples, representing palmitoylated proteins, and total lysate (input) for PSD-95, Gα-i3, and AKAP150. Omission of NH_2_OH before biotinylation resulted in no NeutrAvidin pull down as negative control for non-specific pull down. All lanes are from same blots and exposures but re-arranged to show only relevant lanes. **(D,E)** Quantification of PSD-95 and AKAP150 palmitoylation normalized to respective total inputs. (^∗∗^*p* < 0.01, ^∗∗∗^*p* < 0.001; *t*-test, *n* = 4–9 per condition from at least three independent sets of cultures). Untreated condition was set to equal 100% for each protein.

Removal of PSD-95 from the synapse is likely a major factor in LTD, which manifests itself by removal of synaptic AMPARs from excitatory synapses. PSD-95 palmitoylation is essential for its postsynaptic localization and is modulated by changes in activity, including glutamate receptor activation (El-Husseini Ael et al., 2002; Zhang et al., 2014; Chowdhury et al., 2018). Therefore, we assessed whether palmitoylation of endogenous PSD-95 is altered upon induction of chemical LTD (cLTD) by brief treatment of cultured cortical neurons with NMDA (Lee et al., 1998; Diering et al., 2016). This cLTD is accompanied by dephosphorylation of the AMPAR GluA1 subunit on S845 (Lee et al., 1998; Diering et al., 2016). S845 is an important phosphorylation site for PKA (Roche et al., 1996) and this phosphorylation augments surface expression and postsynaptic functional availability of AMPARs (Joiner et al., 2010; Patriarchi et al., 2018). Furthermore, electrically induced LTD is absent in S845A knock in mice (Lee et al., 2010) and in mice in which association of the protein phosphatase calcineurin with GluA1 via A-kinase-anchoring protein 150 (AKAP150) had been eliminated (Sanderson et al., 2016) indicating that S845 dephosphorylation is required for LTD. After 15 min of washout of NMDA, we observed reduced phosphorylation on S845 of the GluA1 subunit of AMPARs (Figure 1A,B), thus validating this cLTD approach in our cultures. Previous studies used NMDA concentrations ranging from 20 to 75 μM to chemically induce LTD (Lee et al., 1998; Nelson et al., 2013; Diering et al., 2016). We observed a more robust GluA1-S845 dephosphorylation using 50 μM NMDA compared to 20 μM (Supplementary Figures S1A,B) and hence, used this concentration in our experiments with cortical cultures. Moreover, 20 μM NMDA did not significantly reduce surface levels of GluA1 in our hippocampal cultures (Supplementary Figure S1C).

To monitor PSD-95 palmitoylation biochemically, we used the acyl-biotin exchange method (ABE) (Wan et al., 2007; Noritake et al., 2009). Upon cLTD treatment of cultured neurons, the amount of palmitoylated PSD-95 was significantly decreased by ∼30% at 15 min of washout (*p* < 0.01; Figure 1C,D). The reduction in PSD-95 palmitoylation persisted even at 60 min of washout (*p* < 0.001; Figure 1C,D), reflecting the long-lasting nature of LTD. The level of palmitoylation on another palmitoylated protein, Gα-i3, was not affected by the cLTD treatment, showing the specificity of the effect. Omission of hydroxylamine prevented the covalent binding of the thiol-reactive biotin reagent leading to absence of signal in pulldown samples, thus validating specific detection of palmitoylated proteins. AKAP150 is another postsynaptic scaffold protein, which is required for LTD (Lu et al., 2008; Sanderson et al., 2016). A large reduction (>50%) in palmitoylation of AKAP150, was observed in parallel (Figure 1E,F) as seen previously (Keith et al., 2012). However, glycine treatment that is known to induce a form of chemical long-term potentiation (cLTP) in cultured neurons (Liao et al., 2001; Lu et al., 2001) resulted in no significant change in PSD-95 palmitoylation (Supplementary Figure S2). We observed increased phosphorylation on GluA1 S845 with the cLTP treatment, indicating that our treatment was effective.

### Reduction in PSD-95 Palmitoylation in cLTD Requires Ca^2+^/CaM Binding to PSD-95

**FIGURE 2:**
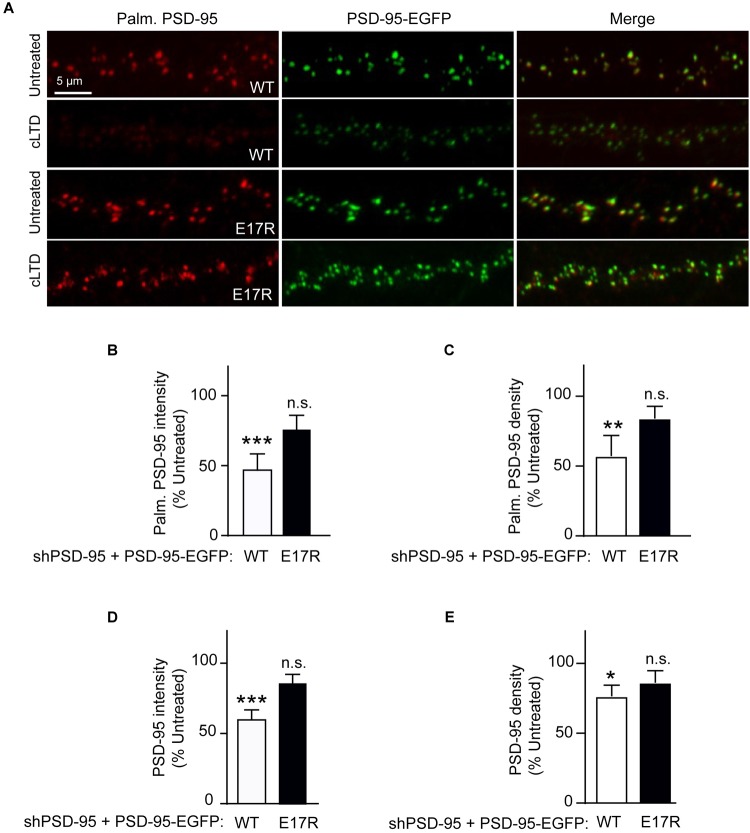
A point mutation of PSD-95 that disrupts Ca^2+^/CaM binding attenuates loss of PSD-95 palmitoylation and synaptic abundance in cLTD. Cultured hippocampal neurons were infected at DIV14 with lentivirus for simultaneous expression of both shRNA against PSD-95 (shPSD-95) and sh-resistant PSD-95-EGFP. In this way, endogenous PSD-95 was replaced with either wild-type (WT) or E17R PSD-95. Cultures at DIV19 were left untreated or treated with NMDA (75 μM) for 5 min followed by washout for 15 min (cLTD) before fixation and staining with hPF11, a recombinant antibody specific for palmitoylated PSD-95. **(A)** Representative confocal microscopic images of dendritic segments showing intrinsic EGFP fluorescence (green) and hPF11 immunostaining (red). **(B,C)** Quantification of hPF11 (Palm. PSD-95) intensity and density showed cLTD-induced reduction in PSD-95 palmitoylation in neurons expressing PSD-95 WT but not in those expressing PSD-95 E17R. **(D,E)** Quantification of GFP intensity (PSD-95) from **(A)** showed cLTD-induced reduction in PSD-95 puncta intensity as well as puncta density in neurons expressing PSD-95 WT but not in those expressing PSD-95 E17R. Dendritic segments from 15 to 20 neurons from 2 to 3 independent sets of cultures were analyzed per condition (^∗∗∗^*p* < 0.001, ^∗∗^*p* < 0.01, ^∗^*p* < 0.05 vs. untreated levels; n.s., not significant; one-way ANOVA followed by Bonferroni’s *post hoc* test). Values were normalized to untreated controls, which were set to equal 100% for each PSD-95 construct.

Stimulation of Ca^2+^ influx through NMDARs upon acute NMDA treatment of mouse brain slices induces binding of Ca^2+^/CaM to PSD-95, which antagonizes PSD-95 palmitoylation (Zhang et al., 2014). Therefore, we hypothesized that the PSD-95- Ca^2+^/CaM interaction underlies reduction in PSD-95 palmitoylation in LTD. To this end, we examined the effect of cLTD treatment on the palmitoylation of the E17R mutant of PSD-95, which shows impaired binding to Ca^2+^/CaM (Chowdhury et al., 2018). To avoid the confounding effects of PSD-95 overexpression, we employed a lentivirus-mediated molecular replacement strategy that involves simultaneous knockdown of endogenous PSD-95 with a validated shRNA and ectopic expression of shRNA-resistant PSD-95 (Schluter et al., 2006; Xu et al., 2008). We had verified a nearly complete loss of endogenous PSD-95 and ectopic expression of GFP-tagged PSD-95 at a level comparable to that of endogenous PSD-95 from uninfected cultures (Chowdhury et al., 2018). To directly visualize the palmitoylated form of PSD-95 within neuronal dendrites, we used a recombinant antibody (clone PF11) that specifically recognizes palmitoylated PSD-95 by immunofluorescence (Fukata et al., 2013). While NMDA treatment resulted in significant reduction in the palmitoylation signal for wild-type PSD-95 (*p* < 0.001, Figure 2A,B), such an effect was clearly attenuated and statistically not significant for the E17R mutant (Figure 2A,B). Similar effects were observed for puncta density measured along the same dendrites (Figure 2C). This finding indicates that Ca^2+^/CaM binding to PSD-95 is required for the reduction in palmitoylation. Notably, the mutation does not affect basal levels of PSD-95 palmitoylation (Chowdhury et al., 2018); thus, the observed decrease in palmitoylated PSD-95 was due to the NMDA treatment and not a reduction in basal PSD-95 palmitoylation or in total postsynaptic PSD-95 the mutation could have caused.

### Ca^2+^/CaM Binding to PSD-95 Mediates Loss of Synaptic PSD-95 in cLTD

PSD-95 palmitoylation is important for its postsynaptic localization. Therefore, to evaluate the functional relevance of the reduced palmitoylation observed in cLTD, we compared the synaptic abundance of wild-type and mutant PSD-95 upon NMDA treatment in parallel to its palmitoylation. Ectopically expressed PSD-95-EGFP shows a highly punctate pattern with discrete clusters along the neuronal dendrite, reflecting almost exclusive accumulation within dendritic spines that mostly represent excitatory synapses (Arnold and Clapham, 1999; Craven et al., 1999; Xu et al., 2008). As a measure of the synaptic PSD-95 content, the EGFP fluorescence intensity of these clusters along the dendrite was quantified. As expected from its effect on PSD-95 palmitoylation, cLTD treatment significantly reduced synaptic levels of wild-type PSD-95 (*p* < 0.001; Figure 2A,D). Importantly, the synaptic levels of the CaM-binding-defective mutant E17R remained largely unaffected with the same treatment, indicating that PSD-95-CaM binding underlies removal of synaptic PSD-95 in cLTD, likely in part by influencing PSD-95 palmitoylation (Zhang et al., 2014; Chowdhury et al., 2018). Puncta density was also reduced for wild-type PSD-95, though to a lesser extent than intensity, upon cLTD treatment while the E17R mutant showed no significant decrease (Figure 2E). Importantly, the E17R mutation does not affect basal levels of PSD-95 synaptic accumulation as well as spine density and size (Chowdhury et al., 2018), ruling out the possibility that the changes in PSD-95 synaptic accumulation observed in LTD are secondary to structural changes induced by the mutation itself. Moreover, the mutation does not alter the basal levels of synaptic NMDARs, measured as GluN1 puncta following surface immunolabeling of neurons expressing the PSD-95 replacement constructs (Supplementary Figure S3). Also, the mutant rescues the decrease in AMPAR-mediated postsynaptic response upon knockdown of endogenous PSD-95 to the same degree as wild-type PSD-95 (Xu et al., 2008).

### Ca^2+^/CaM Binding to PSD-95 Mediates Loss of Synaptic AMPARs in cLTD

**FIGURE 3:**
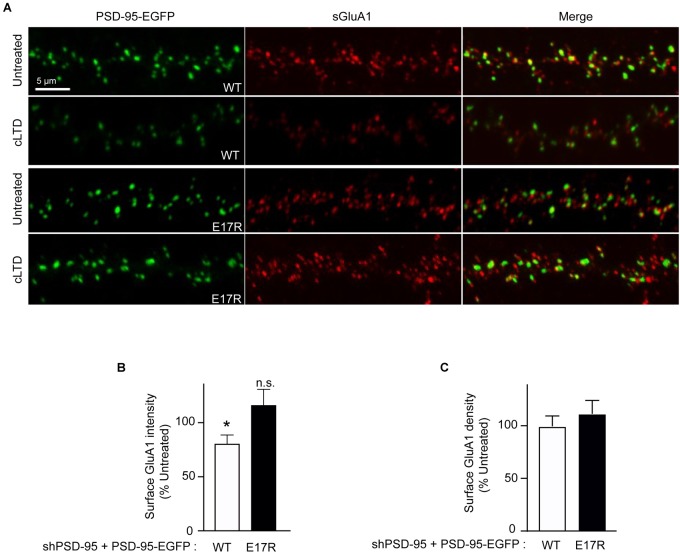
Point mutation of PSD-95 that disrupts Ca^2+^/CaM binding prevents loss of surface AMPARs in cLTD. Cultured hippocampal neurons were infected at DIV14 with lentivirus for simultaneous expression of both shRNA against PSD-95 (shPSD-95) and sh-resistant PSD-95-EGFP to replace endogenous PSD-95 with either wild-type (WT) or E17R PSD-95-EGFP. Cultures at DIV19 were left untreated or treated with NMDA (75 μM) for 5 min or left untreated followed by washout for 15 min (cLTD) before fixation and staining for surface GluA1. **(A)** Representative confocal microscopic images of dendritic segments showing intrinsic EGFP fluorescence (green) and surface GluA1 immunostaining (red). **(B)** Quantification of surface GluA1 intensity from **(A)** showed reduction in AMPAR surface levels upon cLTD treatment in neurons expressing WT PSD-95 but not in those expressing the E17R mutant. Dendritic segments from 12 to 14 neurons from 2 to 3 independent sets of cultures were analyzed per condition (^∗^*p* < 0.05 vs. untreated levels; n.s., not significant; *t*-test). Values were normalized to untreated controls, which were set to equal 100% for each PSD-95 construct. **(C)** Quantification of GluA1 puncta density from **(A)** showed no change upon cLTD treatment.

To determine whether the role of PSD-95-CaM binding in controlling PSD-95 synaptic abundance impacts AMPAR downregulation in cLTD, surface levels of endogenous AMPARs were monitored by labeling with an antibody against the extracellular N-terminus of the GluA1 subunit without permeabilization in primary hippocampal neurons infected with virus carrying either wild-type or mutant forms of PSD-95-EGFP. At 15 min of washout following NMDA treatment, a significant reduction in surface levels of GluA1 was observed when endogenous PSD-95 was replaced with wild-type PSD-95 (*p* < 0.5; Figure 3A,B). In contrast, no such downregulation was observed upon replacement with the E17R mutant. No change in GluA1 puncta density was observed upon cLTD (Figure 3C), which is not unexpected given the relatively modest reduction in puncta intensity. These results show that PSD-95- Ca^2+^/CaM interaction mediates surface AMPAR downregulation seen in cLTD. Taken together, these results demonstrate that interaction of PSD-95 with Ca^2+^/CaM underlies removal of PSD-95 and AMPARs from synapses by reducing PSD-95 palmitoylation.

## Discussion

Our findings provide a mechanistic basis for the removal of synaptic PSD-95 that is essential for AMPAR downregulation in LTD. Upon cLTD stimulation, binding of Ca^2+^/CaM to the PSD-95 N-terminus containing its palmitoylation sites impairs (re)palmitoylation. This binding thereby reduces the synaptic localization of PSD-95, which, in turn, facilitates detachment of AMPARs from postsynaptic sites leading to their eventual loss from cell surface due to internalization. This model is supported by the following lines of evidence: First, palmitoylation of PSD-95 was drastically reduced by 15 min after cLTD stimulation for wild-type PSD-95 when the Ca^2+^/CaM -binding-defective E17R mutant of PSD-95 does not show such a reduction. Second, loss of PSD-95 from the synapses in cLTD was largely blocked by molecular replacement with the mutant PSD-95. Third, depletion of surface AMPARs following cLTD stimulation was absent in presence of the mutant PSD-95.

A number of activity-dependent PTMs regulate synaptic PSD-95 levels and thereby synaptic anchoring sites for AMPARs in LTD. Some of these PTMs pertain to the N-terminal region of PSD-95 as identified in studies investigating the surface stability of AMPARs upon agonist stimulation in dissociated neuron cultures similar to the present study. One study reported that acute NMDA stimulation induced PSD-95 polyubiquitination leading to its proteasomal degradation that depends on a PEST motif in the PSD-95 N-terminus (Colledge et al., 2003). However, using the same treatment in cultured neurons, another study could not detect PSD-95 ubiquitination (Bingol and Schuman, 2004). Like others (Nelson et al., 2013), we did not observe a decrease in total PSD-95 protein levels using acute NMDA stimulation (Figure 1C).

The PEST motif contains the Glu17 residue that we (this study) and others have found to be critical for LTD (Xu et al., 2008). Xu et al. (2008) showed that the E17R mutant of PSD-95 undergoes limited constitutive proteolytic cleavage, which required the PSD-95 residues 53 through 64, and overexpression of this mutant blocks electrophysiologically induced LTD in hippocampal slice cultures. The C-terminal fragment generated in this mutant was proposed to assert a dominant-negative effect on LTD by interfering with crucial C-terminal interactions of PSD-95 with signaling proteins such as AKAP79/150 that are necessary for LTD. However, proteolytic cleavage products of the Q15A and E17R mutant PSD-95 constituted only a fraction of the uncleaved PSD-95 mutants making it difficult to explain how the cleavage products could act in a dominant-negative fashion. Also, the effect of LTD-inducing stimulus on truncation of native PSD-95 was not examined. Whether this mechanism might operate in the physiological context is, thus, not clear. Still, we do not want to exclude the possibility that proteolytic cleavage of PSD-95 contributes to LTD. Yet, our work implicates Glu17 in activity-triggered decline in PSD-95 synaptic abundance thereby reducing the sites for tethering AMPARs at the synapse under cLTD conditions. The resistance of the E17R mutant to NMDA-induced cLTD is based on the critical role of E17 in mediating PSD-95 interaction with Ca^2+^/CaM (Chowdhury et al., 2018). Due to overlapping sites on the PSD-95 N-terminus, binding of Ca^2+^/CaM upon NMDA treatment antagonizes PSD-95 palmitoylation (Zhang et al., 2014). Rapid loss of PSD-95 palmitoylation upon cLTD stimulation, which is largely attenuated in the E17R mutant, provides a molecular mechanism underlying PSD-95 dispersal from the synapse. That Ca^2+^/CaM binding to the PSD-95 N-terminus mediates LTD is also consistent with the finding that the Q15A mutant PSD-95 also prevents LTD (Xu et al., 2008) because this mutation also impairs Ca^2+^/CaM binding to PSD-95 (Chowdhury et al., 2018). Interestingly, Ca^2+^/CaM binding to PSD-95 not only decreases its palmitoylation but also displaces α-actinin from the PSD-95 N-terminus, which is otherwise, like palmitoylation, required for postsynaptic PSD-95 localization (Matt et al., 2018). This mechanism acts most likely in parallel to the reduction in palmitoylation with respect to the activity-induced PSD-95 redistribution.

Palmitoylation at Cys 3 and Cys 5 within the PSD-95 N-terminus is absolutely essential for its synaptic localization (Craven et al., 1999). Palmitoylation is a reversible lipid modification that enables insertion of soluble cytosolic proteins into membranes and dynamically regulates subcellular localization of the target proteins (Salaun et al., 2010). It is widely prevalent among neuronal proteins, including many synaptic proteins, and is a key player in neuronal development and plasticity (Fukata and Fukata, 2010). PSD-95 is the most prevalent of palmitoylated proteins of the synaptic proteome. Treatment of primary hippocampal neurons for several hours with 2-bromopalmitate, an inhibitor of palmitoylation reaction, blocks PSD-95 palmitoylation and disperses synaptic clustering of PSD-95 and AMPARs (El-Husseini Ael et al., 2002). In addition, bath-applied glutamate accelerated the palmitate turnover on PSD-95 and promoted AMPAR endocytosis. Glutamate-induced loss of synaptic AMPARs was blocked by overexpression of a palmitoylation-deficient PSD-95 mutant carrying a C-terminal prenylation motif, that is recruited to the synapse and constitutively membrane-bound, suggesting that palmitate cycling on PSD-95 is required to transduce changes in postsynaptic receptor activation (El-Husseini Ael et al., 2002). Although this early study uncovered a critical role for PSD-95 palmitoylation in regulation of synaptic AMPAR content, whether this phenomenon occurs during LTD that involves downregulation of synaptic AMPARs has not been demonstrated so far. We detected a net decrease in PSD-95 palmitoylation 15 min after acute NMDA treatment, an appropriate cell culture model for LTD, whereas the above study assayed palmitoylation at 1 h of glutamate application. Unlike this previous study (El-Husseini Ael et al., 2002) our results demonstrate that glutamate receptor activation reduces synaptic localization of PSD-95 itself. Although the effect of 2-bromopalmitate on PSD-95 dispersal was previously found to be dependent on Ca^2+^ influx, our study reveals that the reduction in PSD-95 palmitoylation downstream of elevated Ca^2+^/CaM binding to PSD-95 is the mechanistic link between Ca^2+^ influx and PSD-95 redistribution. Moreover, since PSD-95 overexpression by itself increases synaptic AMPAR content as previously observed (El-Husseini et al., 2000; Schnell et al., 2002), the molecular replacement strategy used to substitute endogenous PSD-95 with ectopic PSD-95 in the current study allows reliable assessment of the precise effect of LTD-inducing stimulus. The palmitoylated cysteines on PSD-95 are also nitrosylated upon activation of neuronal nitric oxide synthase (nNOS) following NMDAR stimulation, which inhibits PSD-95 palmitoylation in cerebellar granule cells (Ho et al., 2011). Since NO donors and NOS inhibitors have no effect on hippocampal CA1 LTD (Malen and Chapman, 1997), this phenomenon might not be associated with the cLTD in our hippocampal cultures. Palmitoylation-deficient PSD-95 mutant (C3S,C5S) shows less interaction with the AMPAR auxiliary subunit stargazin in heterologous cells and increased PSD-95 palmitoylation is associated with higher levels of surface AMPARs (Jeyifous et al., 2016), suggesting another mechanism coupling PSD-95 depalmitoylation to AMPAR removal that remains to be tested in synaptic plasticity paradigms.

Additional molecular mechanisms for LTD that converge on PSD-95 synaptic stability have been identified. Dephosphorylation of S295 and increased phosphorylation of T19 on PSD-95 by Glycogen Synthase Kinase 3β have been implicated in postsynaptic PSD-95 localization and LTD (Kim et al., 2007; Nelson et al., 2013). Recent work also identified the death-associated protein kinase 1 (DAPK1) as a novel downstream target of Ca^2+^/CaM activation in LTD (Goodell et al., 2017). We assume that the different molecular events that control PSD-95 in LTD occur in a highly coordinated manner to ensure fine-tuning of AMPARs by synaptic activity. Palmitoylation-dependent PSD-95 synaptic localization is definitely one of the most fundamental means dominating such activity-dependent regulation. In fact, continuous local palmitate cycling on PSD-95 within individual postsynaptic spine defines the precise locus of PSD-95 nanodomains that serve as independent “slots” for anchoring postsynaptic AMPARs (Fukata et al., 2013). Future studies will be forthcoming to gain some more insights into how PSD-95 palmitoylation/depalmitoylation could be regulated in LTD. One potential avenue can be exploring the role of the enzymes catalyzing this reversible lipid modification in LTD.

## Materials and Methods

### Ethics Statement

All animal procedures had been approved by the University of California at Davis and followed NIH guidelines.

### cDNA Constructs

Molecular replacement construct for PSD-95 in lentiviral vector (Schluter et al., 2006) was kindly provided by Dr. Robert C. Malenka (Stanford University, CA, United States).This dual promoter construct contains the human H1 promoter driving expression of a shRNA targeting PSD-95 and an ubiquitin promoter driving expression of a shRNA-resistant PSD-95-EGFP. Glu17 in PSD-95 was mutated to Arg by PCR mutagenesis and mutation was verified by automated DNA sequencing.

### Neuronal Culture and Drug Treatment

Primary hippocampal and cortical neurons were cultured from E18 Sprague-Dawley rat pups as described previously (Perez-Otano et al., 2006; Chowdhury et al., 2018). For immunofluorescence, hippocampal neurons were plated at a density of 10,000–15,000 onto 12-mm coverslips coated with a mixture of poly-DL-ornithine (Sigma) and laminin (Corning), and maintained in Neurobasal medium (Life Technologies) supplemented with 2% (v/v) B27 (Life Technologies), 2 mM GlutaMAX-I (Life Technologies), 1 μg/ml Gentamicin (Life Technologies), and 5% fetal bovine serum (FBS) (Atlanta Biologicals). The anti-mitotic agent 5-fluoro-2’-deoxyuridine was added to the cultures to prevent glial overgrowth. For biochemistry, cortical neurons were plated onto 60 mm dishes coated with poly-L-lysine (Peptide Institute Inc.) at a density of 1 × 10^6^ cells per dish. Cultures were maintained for 17–19 days *in vitro* (DIV) before use. cLTD was induced by bath application of 50–75 μM NMDA in conditioned media for 5 min followed by washout for 15 min in conditioned media. For cLTP, cultures were treated for 5 min with 200 μM glycine in Mg^2+^ -free media in presence of 0.5 μM tetrodotoxin, 20 μM bicuculline, and 1 μM strychnine and then returned to Mg^2+^ -containing media without glycine for 15 min before harvesting.

### Lentivirus Production and Infection of Neuronal Cultures

The lentiviral expression vector and three helper plasmids (pRSV-Rev, pMDLg/pRRE, and VSVG-expressing plasmid) were co-transfected into human embryonic kidney 293T (HEK293T) cells using Ca^2+^ phosphate and maintained in DMEM (Life Technologies) supplemented with 10% FBS. Cell culture media were collected 40 h after transfection, filtered through 0.45 μm filters, and centrifuged at 50,000 × *g* for 2 h at 4^o^C to concentrate the viral particles. To infect neuronal cultures, concentrated viral solutions were added to conditioned media and incubated for 4–6 h before replacing with fresh media.

### Primary Antibodies

Mouse anti-PSD-95 (K28/43) (NeuroMab, UC Davis), rabbit anti-GluA1 (JWH lab; Patriarchi et al., 2016), rabbit anti-Gα-i3 (C10, Santa Cruz Biotechnology), rabbit anti-AKAP150 (Millipore) and rabbit anti-phospho-S845 GluA1 (Cell Signaling) were used for immunoblotting, and rabbit anti-GluA1 (PC246, Calbiochem) and mouse anti-GluN1 (clone 54.1, MAB363, Millipore) for immunofluorescence. The hPF11 antibody was a generous gift from Dr. Masaki Fukata (Okazaki, Japan).

### Immunofluorescence

For surface receptor labeling, cultured neurons at 14 days *in vitro* (DIV) were infected with lentivirus, delivering PSD-95 molecular replacement constructs. Following cLTD treatment, neurons at DIV19 were fixed for 5 min at room temperature in phosphate-buffered saline (PBS) containing 4% paraformaldehyde/4% sucrose, washed with PBS, blocked with 5% bovine serum albumin (BSA)/4% normal goat serum (Jackson ImmunoResearch) in PBS for 30 min, and incubated with anti-GluA1 N-terminal antibody or anti-GluN1 antibody in PBS containing 2% BSA for 2 h at room temperature without permeabilization. Following washes with PBS and blocking for 1 h, samples were incubated with AlexaFluor 555-conjugated species-specific secondary antibody (Molecular Probes) in PBS containing 5% BSA for 1 h. After final washes with PBS, coverslips were mounted onto glass slides using ProLong Gold AntiFade reagent (Molecular Probes). Fluorescence images were acquired on an LSM700 confocal microscope (Zeiss) using a 63× oil-immersion objective (NA 1.4) and optical zoom of 1.5, as a Z-series of 8–10 slices with 0.3–0.4 μm intervals and pixel size 0.1 μm. Neurons with somewhat triangular cell bodies were selected for imaging in order to include pyramidal neurons in our analysis. Neurons with clearly round cell bodies and multiple primary dendrites radiating from the cell body were not used in order to exclude interneurons. All conditions within the same experiment were imaged using the same microscope settings. Maximum intensity projection images were analyzed with MetaMorph Imaging (Molecular Devices). Briefly, 50–70 μm dendritic segments were manually traced and average intensities per square area of surface fluorescence staining measured. For background correction, fluorescence intensities of equivalent areas drawn outside the labeled dendrites were subtracted. Data from several neurons were averaged to obtain a mean. Subsequently, data from 2 to 3 independent experiments performed in different cultures were pooled to obtain a final mean for each condition. Images were acquired and analyzed in a blinded manner.

For labeling palmitoylated PSD-95, cultured neurons were infected with lentivirus and subjected to cLTD treatment as described above. At DV19, neurons were fixed/permeabilized for 10 min in cold (-20^*o*^C) methanol, washed with PBS and after blocking with 1% BSA in PBS for 30 min, were incubated with anti-hPF11 in PBS containing 1% BSA for 2 h at room temperature. Following washes with PBS and blocking for 1 h, samples were incubated with AlexaFluor 647-conjugated anti-human IgG Fc-specific secondary antibody (Jackson ImmunoResearch) in PBS containing 1% BSA for 1 h. After final washes with PBS, coverslips were mounted and imaged as described above. Average fluorescence intensities per square area for the total PSD-95-GFP signal as well as the hPF11 signal were determined within GFP-positive puncta along 50–70 μm dendritic segments. Data from several neurons were averaged to obtain a mean for each condition. Subsequently, data from 2 to 3 independent experiments performed in different cultures were pooled to obtain a final mean for each condition.

### Palmitoylation Assay (ABE Method)

Palmitoylation was assessed in cell extracts from sister cultures of DIV 17–18 cortical neurons using acyl-biotinyl exchange (ABE) method as described previously (Wan et al., 2007) with certain modifications. After brief rinse with PBS, cells were lysed in 0.1 ml of lysis buffer (LB; PBS, pH 7.2, 150 mM NaCl, 5 mM EDTA, 1.5 μM pepstatin A, 2.1 μM leupeptin, 0.3 μM aprotinin, 0.2 mM PMSF, 10 mM NaF) containing 2% SDS and, to block free thiols, 25 mM N-ethyl-maleimide (NEM; Sigma). After 15 min of extraction at 37^o^C, samples were diluted 10-fold with LB containing 2% Triton X-100 and 25 mM NEM and incubated at 4^o^C for 1 h. Lysates were cleared by centrifugation at 16,000xg for 10 min and resulting supernatants were used for the ABE assay after estimating protein concentrations using the BCA assay (ThermoScientific). Proteins in supernatants (140-150 μg) were precipitated by chloroform-methanol (CM) and protein pellets solubilized in 0.5 ml of PBS, pH 7.2, 4% SDS, 5 mM EDTA (4SB) supplemented with 25 mM NEM at 37^o^C for 10 min. The protein was diluted five-fold with LB containing 0.2% Triton X-100 and 1 mM NEM and incubated at 4^o^C overnight with end-to-end rotation. Excess NEM was removed by three sequential CM precipitations followed by solubilization in 0.24 ml of buffer 4SB. To cleave thioester bonds and allow incorporation of a biotin moiety at exposed sulfur atoms, each sample was diluted five-fold in 0.7 M hydroxylamine (NH_2_OH), pH 7.4, 0.2% Triton X-100, 1.5 μM pepstatin A, 2.1 μM leupeptin, 0.3 μM aprotinin, 0.2 mM PMSF (HB) containing 1 mM EZ-link HPDP-biotin (ThermoScientific). As a control, a duplicate of one of the samples was diluted five-fold in the same buffer in which hydroxylamine was replaced with Tris (50 mM, pH 7.4). In absence of hydroxylamine, palmitate groups are not removed thereby preventing biotinylation-mediated purification. The mixture was incubated at room temperature for 1 h with end-to-end rotation and subjected to CM precipitation. The precipitated protein was solubilized in buffer 4SB, diluted five-fold with buffer LB containing 0.2% Triton X-100 and 0.2 mM HPDP-biotin, and incubated for 1h at room temperature with end-to-end rotation. Unreacted HPDP-biotin was removed by two sequential CM precipitations, and the protein pellets solubilized in 0.1 ml of PBS, pH 7.2, 2% SDS, 5 mM EDTA (2SB). Following two-fold dilution in LB containing 0.2% Triton X-100, 10% (v/v) of each sample was saved as input to assess total expression levels. Samples were further diluted five-fold in LB containing 0.2% Triton X-100 and incubated at room temperature for 30 min with end-to-end rotation. After brief centrifugation, the supernatant was incubated with 40 μl UltraLink NeutrAvidin Plus agarose beads (ThermoScientific) for 90 min at room temperature with end-to-end rotation to isolate biotinylated proteins. After washing the beads with LB containing 0.2% Triton X-100 and 0.2% SDS, bound proteins were eluted with 50 μl of SDS-PAGE sample buffer at 95^o^C for 5 min. Eluates and inputs were subjected to SDS-PAGE and analyzed by Western blotting using indicated primary antibodies and horseradish peroxidase-conjugated secondary antibodies (Bio-Rad) before ECL detection (SuperSignal West Femto, ThermoScientific). Films were scanned and non-saturated, immunoreactive bands were quantitated using Adobe Photoshop. The level of palmitoylation for each protein was quantified as the ratio obtained by normalizing the band in the pulldown blot representing the palmitoylated protein to the corresponding input band representing the total protein for each condition. Data from at least 3 independent experiments performed in different cultures were pooled to obtain a final mean for each condition.

### Statistical Analysis

All data were expressed as mean ± SEM. Student’s unpaired two-tailed t test was used for comparing two groups. Multiple comparisons were performed using one-way ANOVA followed by Bonferroni’s posthoc test for pairwise comparisons. Statistical significance was determined with Graphpad Prism and was considered as *p* < 0.05.

## Data Availability

The datasets generated for this study are available on request to the corresponding author.

## Author Contributions

DC and JH designed the study and wrote the manuscript. DC performed the experiments and analyzed the data.

## Conflict of Interest Statement

The authors declare that the research was conducted in the absence of any commercial or financial relationships that could be construed as a potential conflict of interest.
