# Supplementary material for: Ca2+/Calmodulin Binding to PSD-95 Downregulates Its Palmitoylation and AMPARs in Long-Term Depression
Source: Front Synaptic Neurosci. 2019 Mar 12;11:6. doi: 10.3389/fnsyn.2019.00006 (PMC6422948; doi:10.3389/fnsyn.2019.00006)
Supplement: Supplementary file 1 [file Data_Sheet_1.PDF]

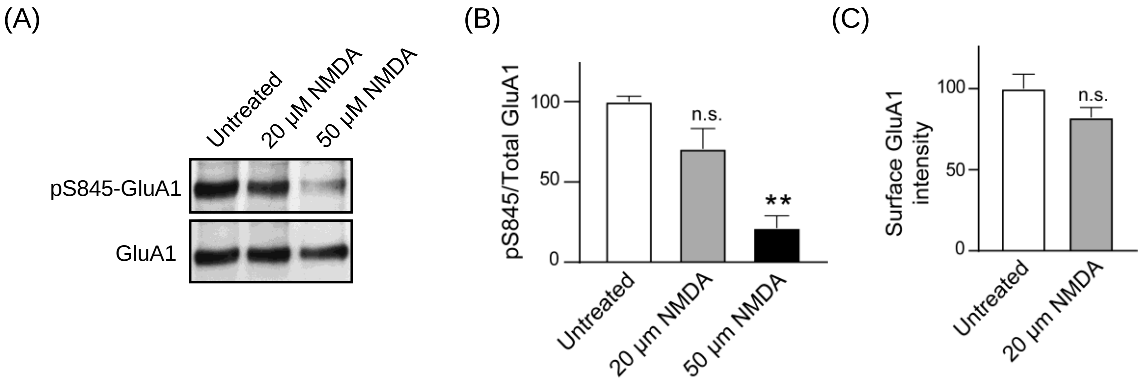

Supplementary Figure 1. Effect of NMDA dosage on AMPAR phosphorylation and surface expression. Cultured neurons, cortical (A-B) or hippocampal (C), were either left untreated or treated with NMDA (20 or 50 µM) for 5 min followed by washout for 15 min. (A) Total cell lysates were analyzed by immunoblotting and probed sequentially with a phospho-specific antibody against GluA1 S845 and total GluA1. Representative immunoblots are shown. (B) Quantification of GluA1 S845 phosphorylation normalized to total GluA1 levels per condition. (**$p<0.01$ vs untreated; n.s., not significant, one-way ANOVA followed by Bonferroni's *posthoc* test, $n=3$). Untreated condition was set to equal 100%. (C) Neurons were fixed and stained for surface GluA1. Quantification of surface GluA1 intensity upon NMDA treatment (17-18 neurons from 2 independent experiments were analyzed per condition, unpaired t-test).
