# Supplementary material for: Ca2+/Calmodulin Binding to PSD-95 Downregulates Its Palmitoylation and AMPARs in Long-Term Depression
Source: Front Synaptic Neurosci. 2019 Mar 12;11:6. doi: 10.3389/fnsyn.2019.00006 (PMC6422948; doi:10.3389/fnsyn.2019.00006)
Supplement: Supplementary file 2 [file Data_Sheet_2.PDF]

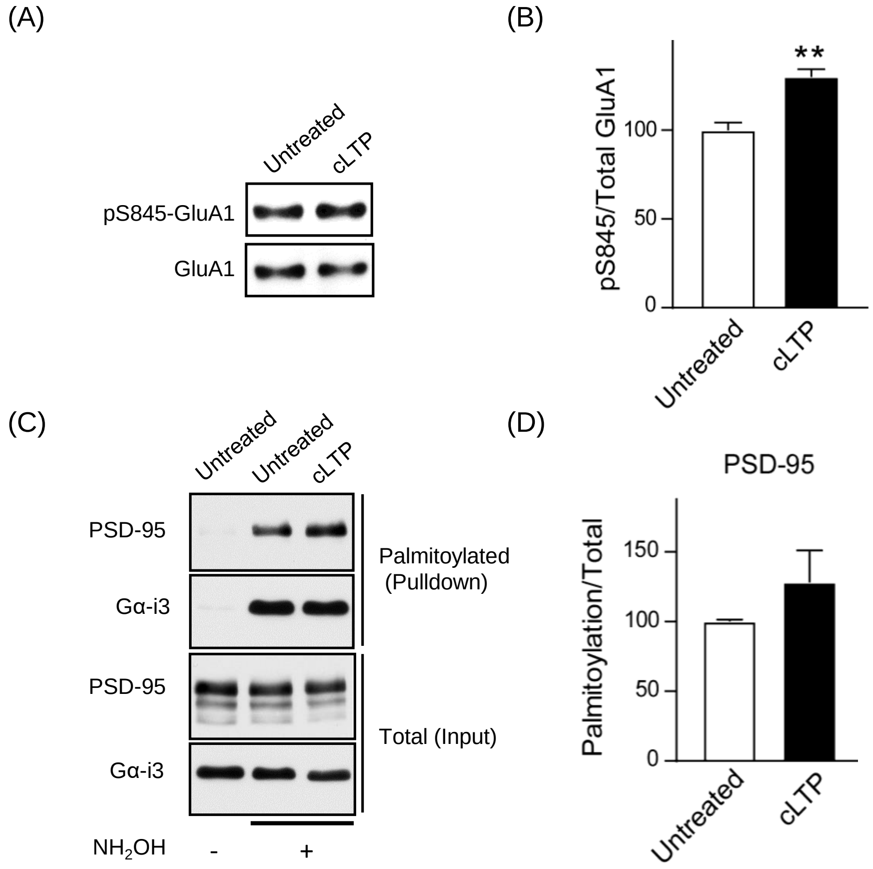

Supplementary Figure 2. Effect of cLTP treatment on PSD-95 palmitoylation. Cultured cortical neurons were either left untreated or treated with glycine (200 µM) for 5 min followed by washout for 15 min (cLTP) before harvesting, extraction and analysis of palmitoylation by ABE method and pulldown with NeutrAvidin agarose beads. (A) Total lysates were analyzed by immunoblotting to examine levels of S845 phosphorylation by immunoblotting with a phospho-specific antibody against phosphoS845. After stripping, the same blot was reprobed with an antibody against total GluA1. Representative immunoblots are shown. (B) Quantification of GluA1 S845 phosphorylation normalized to total GluA1 levels per condition. (**p<0.01; t-test, n=3 per condition). Untreated condition was set to equal 100%. (C) Representative immunoblots of NeutrAvidin pulldown samples, representing palmitoylated proteins, and total lysate (input) for PSD-95 and Gα-i3 are shown. Omission of NH_2OH before biotinylation resulted in no NeutrAvidin pull down as negative control for non-specific pull down. (D) Quantification of PSD-95 palmitoylation normalized to total levels. (n=5-6 per condition). Untreated condition was set to equal 100%.
