# Supplementary material for: Ca2+/Calmodulin Binding to PSD-95 Downregulates Its Palmitoylation and AMPARs in Long-Term Depression
Source: Front Synaptic Neurosci. 2019 Mar 12;11:6. doi: 10.3389/fnsyn.2019.00006 (PMC6422948; doi:10.3389/fnsyn.2019.00006)
Supplement: Supplementary file 3 [file Data_Sheet_3.PDF]

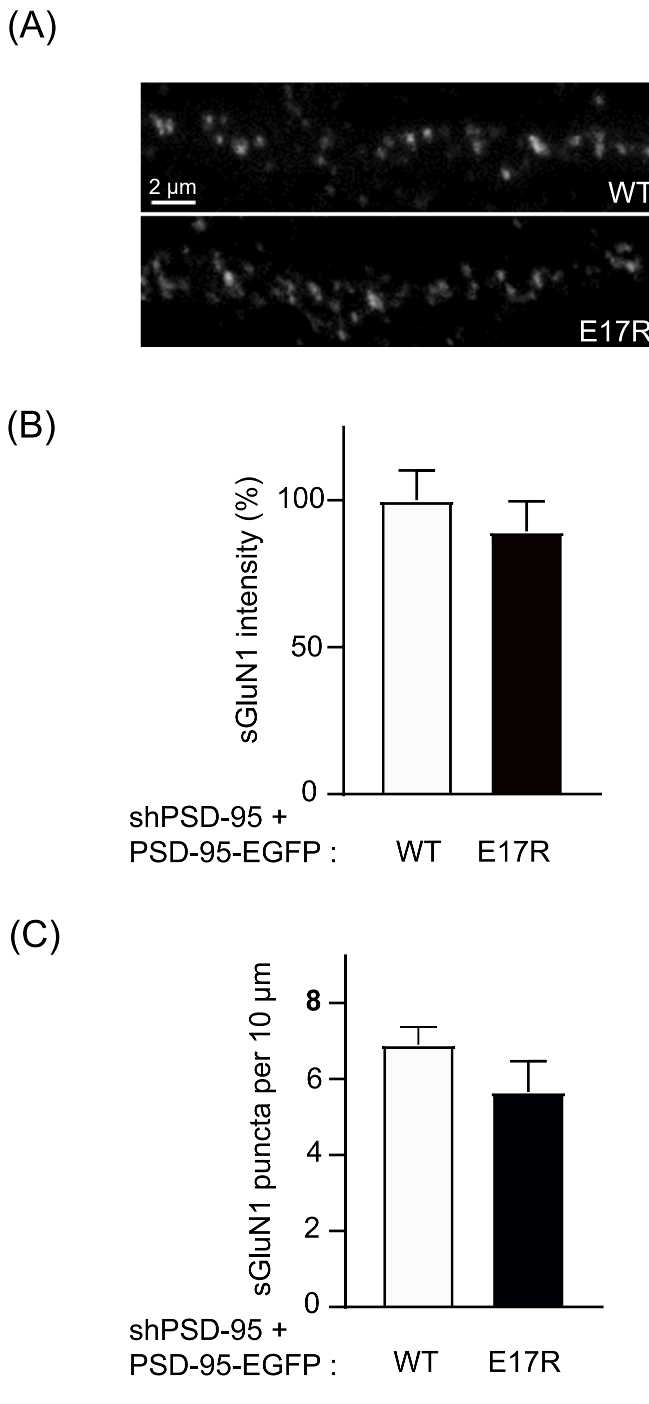

Supplementary Figure 3. Effect of PSD-95 mutation on surface NMDAR levels. Cultured hippocampal neurons were infected at DIV14 with lentivirus for simultaneous expression of both shRNA against PSD-95 (shPSD-95) and sh-resistant PSD-95-EGFP to replace endogenous PSD-95 with either wild-type (WT) or E17R PSD-95-EGFP. Cultures at DIV19 were fixed and stained for surface GluN1. (A) Representative confocal microscopic images of dendritic segments showing surface GluN1 immunostaining. (B-C) Quantification of surface GluN1 intensity and puncta density from (A) showed no significant difference between WT and E17R (n=8-10 neurons per condition, t-test).
